# Wall shear stress and its role in atherosclerosis

**DOI:** 10.3389/fcvm.2023.1083547

**Published:** 2023-04-03

**Authors:** Manli Zhou, Yunfeng Yu, Ruiyi Chen, Xingci Liu, Yilei Hu, Zhiyan Ma, Lingwei Gao, Weixiong Jian, Liping Wang

**Affiliations:** ^1^ College of Traditional Chinese Medicine, Hunan University of Chinese Medicine, Changsha, China; ^2^ The First Affiliated Hospital of Hunan University of Chinese Medicine, Changsha, China; ^3^ National Key Discipline of Traditional Chinese Medicine Diagnostics, Hunan Provincial Key Laboratory, Hunan University of Chinese Medicine, Changsha, China; ^4^ College of Rehabilitation Medicine and Health Care, Hunan University of Medicine, Huaihua, China

**Keywords:** wall shear stress, atherosclerosis, endothelial cells, haemodynamic factors, biological factors, CFD

## Abstract

Atherosclerosis (AS) is the major form of cardiovascular disease and the leading cause of morbidity and mortality in countries around the world. Atherosclerosis combines the interactions of systemic risk factors, haemodynamic factors, and biological factors, in which biomechanical and biochemical cues strongly regulate the process of atherosclerosis. The development of atherosclerosis is directly related to hemodynamic disorders and is the most important parameter in the biomechanics of atherosclerosis. The complex blood flow in arteries forms rich WSS vectorial features, including the newly proposed WSS topological skeleton to identify and classify the WSS fixed points and manifolds in complex vascular geometries. The onset of plaque usually occurs in the low WSS area, and the plaque development alters the local WSS topography. low WSS promotes atherosclerosis, while high WSS prevents atherosclerosis. Upon further progression of plaques, high WSS is associated with the formation of vulnerable plaque phenotype. Different types of shear stress can lead to focal differences in plaque composition and to spatial variations in the susceptibility to plaque rupture, atherosclerosis progression and thrombus formation. WSS can potentially gain insight into the initial lesions of AS and the vulnerable phenotype that gradually develops over time. The characteristics of WSS are studied through computational fluid dynamics (CFD) modeling. With the continuous improvement of computer performance-cost ratio, WSS as one of the effective parameters for early diagnosis of atherosclerosis has become a reality and will be worth actively promoting in clinical practice. The research on the pathogenesis of atherosclerosis based on WSS is gradually an academic consensus. This article will comprehensively review the systemic risk factors, hemodynamics and biological factors involved in the formation of atherosclerosis, and combine the application of CFD in hemodynamics, focusing on the mechanism of WSS and the complex interactions between WSS and plaque biological factors. It is expected to lay a foundation for revealing the pathophysiological mechanisms related to abnormal WSS in the progression and transformation of human atherosclerotic plaques.

## Introduction

**Figure 1:**
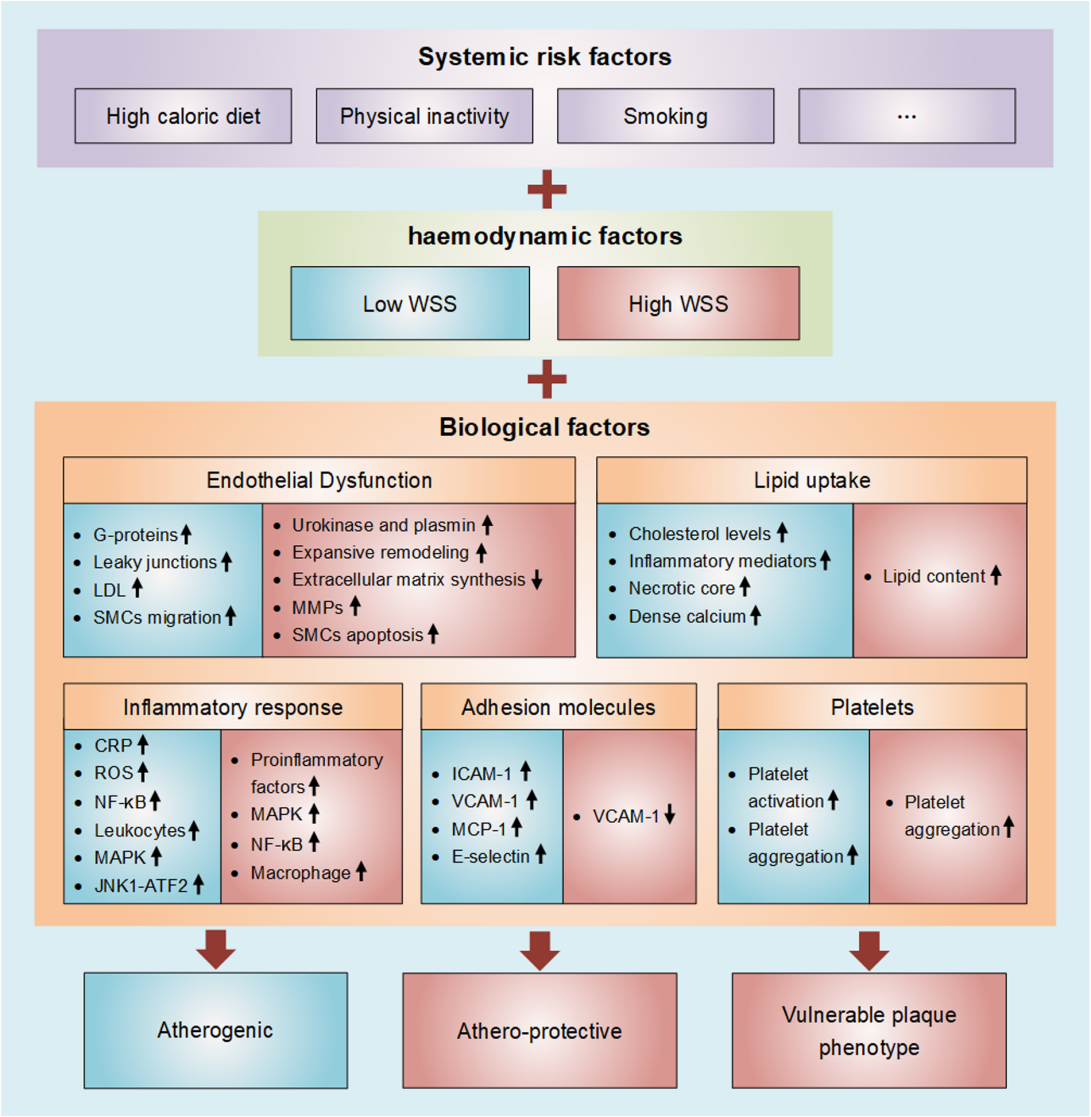
Diagram of wall shear stress involved in atherosclerosis. Atherosclerosis is thought of as subject to a triad of, and especially interactions among, systemic risk factors, haemodynamic forces, and the biological response of the wall. Atherosclerosis is characterized by progressive endothelial cell injury, arterial inflammation, lipid deposition, platelet activation, and the accumulation of extracellular matrix in the arterial wall. These biological factors are all strongly influenced by WSS (Blue represents low WSS and red represents high WSS).

Atherosclerosis (AS) is a local, multifactorial, complex disease, and the leading cause of death worldwide. Sometimes, there is evidence of atherosclerosis, but there are no specific clinical symptoms of atherosclerotic stenosis (1), which means that stenosis is not the key factor to evaluate atherosclerosis. Atherosclerosis is thought of as subject to a triad of, and especially interactions among, systemic risk factors, haemodynamic forces, and the biological response of the wall (2). Atherosclerosis is related to systemic risk factors, which can be aggravated by lifestyle factors such as high caloric diet, physical inactivity, and smoking, but atherosclerosis is, in a sense, a geometrically focal disease (3, 4). Plaque progression and eventually plaque rupture is influenced by a complex interaction between biological and haemodynamic factors (5). The initiation of plaque formation has been strongly linked to a hemodynamic parameter: wall shear stress (WSS) (4). WSS is the frictional force exerted by flowing blood on the vessel wall, which significantly influences the evolution of atherosclerotic disease (6). Vascular endothelial cells (VECs) are in direct contact with flowing blood, therefore sense different blood flow patterns and bear most of the WSS (7). Atherosclerosis is characterized by progressive endothelial cell injury, arterial inflammation, lipid deposition, platelet activation, and the accumulation of extracellular matrix in the arterial wall (8). These biological factors are all important in atherosclerosis, and they are all strongly influenced by WSS, albeit with different mechanisms. The shear force exerted on the ECs determines the permeability of these cells to certain biochemicals. While the biochemical flux is controlled by the WSS magnitude, the localization of biochemicals near the wall is governed by the WSS topological skeleton (9). Atherosclerotic plaques form at predetermined locations where WSS is low and/or oscillatory, and initial plaque growth is generally accompanied by outward remodeling. with the development of the disease, however, plaques will begin to invade the lumen, thus affecting local hemodynamics. Upstream and at the throat of the plaque, WSS levels are high, while low WSS is found downstream (10). In general, in the plaque initiation process low and oscillatory shear stress is considered atherogenic, whereas high shear stress is athero-protective (11). Upon further progression of plaques, high WSS is associated with the formation of vulnerable plaque phenotype. Simply considering systemic risk factors might give a first indication on risk of plaque, but the interplay with haemodynamic factors and biological factors should be taken into consideration. Computational fluid dynamics (CFD) is the most reliable methods to assess patterns of WSS at present, and this technology has commonly been employed (12). Combining the imaging system with the WSS quantitative analysis based on CFD in the future can enable better prediction of the occurrence and development of atherosclerosis by using non-invasive medical imaging techniques for the early diagnosis of atherosclerosis. In this review, we summarize the mechanism of WSS in the pathological evolution of atherosclerosis from these three aspects, and focus on the complex interaction between WSS and plaque biological factors. As shown in Figure 1.

### The role of low/high WSS in AS

In each cardiac cycle, the interactions of pulsatile blood flow with arterial geometries generate complex biomechanical forces on the vessel, including multiple mechanical forces that are exerted on the vessel wall (axial, circumferential forces) or on the endothelial surface (shear stress) (5). Axial forces is difficult to measure *in vivo* experiments, and circumferential forces studies are relatively rare in the medical field, resulting in the first two forces contributing relatively poorly characterized to atherosclerosis compared with shear stress. Studying the biological significance of shear stress may have clinical implications in arteries subject to complicated flow patterns. Shear stress is described mathematically for ideal fluids, and is thought to affect only the inner surface of the vessels (13). WSS is generated by the friction of blood flow on the endoluminal surface of vessel based on its anatomical characteristics, such as vessel diameter, curvature and bifurcation, as well as flow profile, especially around the vessel surface (14). For non-Newtonian fluids, shear stress (*τ*) is defined by the Ostwald de Waele equation: $\tau \;=\;\kappa \;\times \;\dot{\gamma }n$, where γ is the shear rate, n and k are adhesion. For Newtonian fluids flowing upon a planar surface, shear stress is determined according to Newton's law by the equation: *τ *= µ·du/dy, where μ is the kinetic viscosity, u the fluid velocity, y the distance from the surface and du/dy the shear rate (15). In the ideal state of straight vessels, the magnitude of shear stress can be estimated to be directly proportional to blood flow and viscosity, and inversely proportional to the cubic power of vessel diameter (11). Understanding the complex fluid dynamics of arteries will contribute to the future simulation-based CFD studies, especially in the context of atherosclerosis (16). Because of the limited time or space resolution of the imaging systems, examining the atherosclerosis in details *in vivo* process has been difficult (17). Regular observation of arterial geometry with imaging system is unable to evaluate the susceptibility of atherosclerosis, but CFD would be useful to predict it, and WSS can be used as one of the effective parameters for early diagnosis of atherosclerosis (17). Several studies have applied CFD to simulate and analyze the WSS (18). To calculate the characteristics of WSS through CFD modeling, first of all, the complex 3D geometry of the arteries should be reconstructed based on clinical imaging data, including intravascular imaging cross sections and biplane angiography. Then, the 3D geometry is divided into reasonable grids to refine key focus areas and improve the resolution of CFD analysis results. With the blood and blood vessel characteristics of the patient as the boundary conditions of the model input, the WSS distribution characteristics of the inner wall of the blood vessel are finally calculated iteratively. Boundary conditions can be “experimentally” manipulated during the experiment to determine their effect on the WSS distribution within the artery. Thus, using reconstructed vascular geometry, CFD modeling can be used to mathematically calculate WSS using data obtained by different imaging modalities. With the continuous improvement of the computer performance-cost ratio and the ready-made availability of the technology for incorporating image-derived geometries into CFD modeling, this method is more reliable than the method based solely on *in vivo* measurement (12). Riedl et al. (19) computed WSS from MRI data, demonstrating that low WSS was associated with an increased local plaque burden. IVUS-derived CFD and palpography were used for WSS calculation and plaque strain analysis, which is completed by Gijsen et al. (20). Suo et al. (21) used computer tomography (CT) to describe the geometry of mouse aorta and determined its WSS through CFD calculation. Hetterich and colleagues reported the feasibility of applying CFD techniques to coronary computed tomography angiography for determining WSS (22).

The spatial distribution of human arterial plaque components is heterogeneous, and traditional risk factors do not fully explain the focal aggregation of plaque, in which shear stress is thought to play an important role (23). To study effects of shear stress *in vivo*, several investigators have established animal models of low WSS and high WSS. A commonly used model of low shear stress is produced by ligating the outflow branches of the carotid artery to decrease blood flow and thus the shear stress. The construction methods of high shear stress model include forming an arteriovenous fistula in the carotid or femoral arteries, either in rabbits or rats, ligating one of the carotid arteries to increase the flow and shear stress of the contralateral carotid artery, or using the transposition of a vein graft into the arterial circulation (13). The low and/or oscillatory WSS hypothesis has become the consensus mechanism for the initiation of atherosclerosis (24). There is evidence from both cross-sectional and longitudinal human studies to suggest that high WSS is frequently associated with high-risk plaque features (25). Plaque initiation usually occurs in the low shear stress region, and atherosclerosis usually shows preferential localization characteristics in the low WSS region (26). WSS having low average magnitude and large oscillatory changes during the cardiac cycle is recognized as an atheroprone haemodynamic phenotype (11). Low and oscillatory WSS creates a pro-atherogenic environment for lesion development (27). Exposure to low and oscillatory WSS is a significant independent risk factor for identifying individuals at greater susceptibility for atherosclerosis (28). In the study of Mahmoudi, subject-specific CFD simulations were performed in ten coronary artery models of diseased and healthy subjects, and the results showed that low WSS magnitude promoted atherosclerosis by increasing atherogenic biochemical localization (29). Stone et al. (30) published the largest study on the effect of WSS on human coronary plaque growth. They found that low WSS was an independent predictor of increased plaque load and lumen obstruction over a 6-month period, which could help identify arterial segments prone to progressive plaque leading to lumen stenosis as early as possible in the clinical environment. Hoogendorn et al. (31) performed coronary artery imaging at three time points (3 (T1), 9 (T2) and 10–12 (T3) months) in adult familial hypercholesterolemic pigs that were fed a high-fat diet. They proved that the occurrence and development of plaque were related to low and oscillatory WSS. The largest plaque growth was found in sectors with T1 low time-averaged WSS (TAWSS) that changed to higher TAWSS at T2 due to lumen intrusion of large plaques. The plaque growth rate during plaque progression between T2-T3 was lowest of all analysed sectors. A two-dimensional ultrasound study in 48 patients also showed plaque progression in low WSS areas during a 12-year follow-up (32). Plaque progression is accompanied by compensatory dilated remodeling of the vessel to maintain its lumen diameter, and outward remodelling will lead to apersistence of low shear stress, thereby exaggerating lipid uptake and inflammation (5). In a study of P.H. Stone et al. using intravascular ultrasound, the increase in the volume of atheroma was significantly more frequent in coronary artery segments with low WSS (33). Once the atherosclerotic plaque invades the lumen, vascular endothelial cells undergo changes in local shear stress (30). The highest WSS values were found at the minimum lumen area of the coronary lesions, while the lowest WSS values were found in the distal segment of the plaque, i.e., high shear stress in the upstream portion of the plaque and low shear stress in the downstream portion of the plaque (34).

High WSS prevents atherosclerosis *via* mechanotransduction pathways. As the effective oxygen transport is from the lumen into the wall, it tends to localize with regions where the near-wall normal velocity is towards the wall, which increases the oxygen flux in the higher WSS values. From a transport point of view, high WSS increases the production of NO and ATP, resulting in a localized elevation in oxygen flux, and reduces near-wall localization of atherogenic factors such as LDL and monocytes (29). while high WSS prevents atherosclerosis growth, at the same time it can promote plaque vulnerability and rupture for established plaques (35). Plaque rupture is most often encountered at the high WSS–exposed upstream site (36). This region is usually the thinnest region of the fibrous cap (37) and is co-located with increased macrophage density (38), intraplaque hemorrhage (39), and local microcalcifications (40). Samady et al. (41) found increased plaque vulnerability in areas with high shear stress plaques in twenty patients with coronary artery disease at 6-month follow-up. In a single-center, retrospective observational study conducted by Kojima et al. (17), 45 consecutive patients with confirmed or suspected coronary artery disease underwent 3D-CT as well as coronary angiography and non-obstructive general angioscopy (NOGA) for the aortic arch between 2015 and 2019. The purpose of this study is to explore the relationship between aortic NOGA-derived ruptured plaque (RP) and the stereographic distribution of WSS by CFD modeling using three-dimensional computed tomography (3D-CT) angiography. The results showed that aortic RP detected by NOGA was strongly associated with a higher maximum WSS in the aortic arch derived by CFD using 3D-CT. Their findings support the previous research results that the high WSS value increases the risk of RP. Fukumoto et al. (42) also believe that localized high WSS is a trigger of fibrous cap rupture, which involves a spiral catastrophic cascade leading to RP. Similar results were obtained in studies of Corban et al. and Murata et al. (43, 44). Atherosclerosis exposed to high WSS results in up-regulation of VSMCs apoptosis, up-regulation of macrophages and the increase in the activity of matrix metalloproteinases, which lead to thinning of plaque cap, its erosion or rupture (45). In summary, low WSS was associated with plaque localization and clinical luminal stenosis. Plaque invasion into the vascular lumen produces locally high WSS regions located upstream as well as low WSS regions located downstream. High WSS is potentially important for identifying plaque locations with increased risk of plaque rupture (46).

The complex hemodynamic environment can be only partially described by WSS, which stimulates a proliferation of haemodynamic parameters (47). WSS topological skeleton has provided a new theory for the role of WSS in regulating near-wall biotransport processes in cardiovascular flows, reflecting the presence of blood flow features associated to adverse vascular response (48). The CFD method is mainly used to test the WSS topological skeleton features based on Lagrangian or Eulerian. It has been proved that the combination of WSS topology (WSS directionality and manifolds) as well as WSS magnitude can completely explain the biochemical and cell localization patterns in atherosclerosis (29). The attracting WSS Lagrangian coherent structures (LCS) (unstable manifolds) lead to near-wall accumulation of biochemicals, while the repelling WSS LCS (stable manifolds) tend to push away biochemicals by forming near-wall transport barriers (29). The models utilized to simulate the near-wall transport of biological factors were provided to visualize the near-wall velocity patterns of WSS streamlines. They compared the diseased arteries with healthy swine LAD arteries and found that the presence of plaque results in more complex WSS topological features. These topological features around the plaque showed multiple fixed-points and complex WSS LCS patterns (29). Generally, plaques create backward and attracting WSS patterns downstream of the plaque, attracting atherogenic biochemicals like LDL (49). However, it should be noted that atheroprotective biochemicals such as NO and ATP also localize in similar regions. It is not clear how the combination of these competing effects play out in the process of atherosclerosis. Morbiducci et al. (28) conducted personalized computational hemodynamic simulations on a cohort of 13 carotid models pre-carotid endarterectomy (CEA) and at 1 month after CEA, and analyzed the WSS topological skeleton using a Eulerian method based on the WSS vector field divergence. The study confirmed the direct relationship between WSS topological skeleton and vascular disease markers. The WSS topological skeleton features here considered could contribute to promote long-term restenosis, which represents recurrent atherosclerosis.

### Effect of WSS on different types of cells in blood vessels

Vascular endothelial cells are located in the vessel wall and are subject to hemodynamic effects. Endothelial cells not only sense shear stress, but also respond specifically to different types of shear stress (7). The endothelial structure and integrity of healthy arteries exposed to physiologic WSS remained intact, with endothelial cells arranged in elongated spindles parallel to the direction of blood flow, and little cell turnover (8). WSS in normal physiological range has a protective effect on endothelial cells, and its mechanism involves local anti-inflammatory effect, reducing the expression of adhesion molecules and inflammatory mediators, and preventing apoptosis of endothelial cells, which forms an anti-atherogenic molecular environment to a large extent (50). Endothelial cells are sensors of wall shear stress. WSS not in normal physiological range is the key inducement of endothelial injury (43, 51). When ECs are subjected to forces such as wall shear stress (WSS) and mechanical stretch, their functions, activities, integrity, as well as their phenotype might be altered, resulting in physiological and pathophysiological changes in blood vessels (52). In vitro and *in vivo* studies have shown that endothelial cells respond to shear force changes by altering their morphology, proliferation, and gene expression (6), thereby inducing an atherogenic endothelial phenotype (51), promoting lipid accumulation and oxidation, inflammatory cell infiltration, smooth muscle cell proliferation and extracellular matrix production (53). It is well established that low and oscillatory WSS activates a series of responses in ECs (11). Shear stress affects the cellular polarization and alignment of ECs *via* activating the small G-proteins at focal adhesions and forming the stress fibers network, while stress fiber networks under low and oscillatory forces promote atherosclerosis (29). The permeability of endothelial intercellular clefts is not constant and it changes in response to WSS and biochemical signaling. In vitro studies showed that the number of leaky junctions in cultured ECs increased in low WSS regions, which promoted the the permeability of ECs towards LDL macromolecules (54). Mahmoudi et al. (29) found that the LDL concentration patterns show spotty elevation in regions with low WSS magnitude, and these spotty accumulations are related to regions with more permeability of ECs due to leaky junctions. Response to oscillatory shear is generally the same as for static conditions, including greater cell turnover, which increases macromolecular transport through gaps in the monolayer (55). High WSS can induce vascular endothelial cells to produce urokinase and plasmin, which can effectively activate metalloproteinases, thus greatly increasing the instability of fiber cap structure (56, 57). Mattsson et al. (58) also believed that high shear stress could stimulate endothelial cells and lead to thinning of the fibrous cap. Together, these processes regulate the structure and function of endothelial cells, influence the surrounding cellular environment, and change the balance between inhibiting and promoting atherosclerotic processes (8). Vascular smooth muscle cells (VSMCs) also respond directly or indirectly to shear stress, leading to regulation of cell proliferation, migration, and differentiation between contractile and secretory phenotypes, with the vessel wall continuously fine-tuning its activity in response to shear stress (59). Normally, VSMCs are not affected by the mechanical forces of blood flow. However, under relevant pathological conditions, due to increased endothelial apoptosis and neointima formation, the VSMCs and collagen fibre proliferate in great quantities and the arterial elasticity drops (60). Some researchers used ultrasound linear array probe for scanning the rabbit carotid arteries to obtain the WSS and the elasticity values in the atherosclerotic arteries. Their study has confirmed that with the development of atherosclerosis, the decrease of arterial WSS was negatively correlated with the increase of arterial wall elasticity, which means that as the arterial WSS decreases the arterial wall becomes less elastic (60). Because the elasticity value is a sensitive index in the process of atherosclerotic fibroplaques formation, WSS combined with wall elasticity is helpful to improve diagnostic accuracy of atherosclerosis. In addition, low WSS can stimulate the migration of SMCs from the middle layer to the inner membrane, and mediate the excessive proliferation and growth of the vascular wall region with low WSS, leading to vascular stenosis or contractile remodeling, and finally forming thin fibrous cap plaques (61). In contrast, areas exposed to high WSS tend to undergo expansive remodeling, and once reaching the limit of expansive remodeling, further plaque growth will invade the lumen. High WSS-mediated plaque destabilization ultimately inhibits extracellular matrix synthesis and upregulates matrix metalloproteinases (MMPs) synthesis by macrophages through altered expression of SMCs, which degrades the intravascular elastic lamina and induces SMCs apoptosis, leading to arterial dilation, a set of changes that may enhance plaque vulnerability (41). In addition, data from human carotid autopsy studies also suggest that high WSS coexists with increased macrophage levels and plaque rupture (62). Schaar et al. (63) used intravascular ultrasound-palpography to probe coronary arteries and showed that high WSS regions contained fewer SMCs and higher lipid and macrophage content compared to low WSS regions.

### Effect of WSS on lipid uptake in vascular wall

Atheromatous plaque formation is associated with lipid accumulation in the vessel wall (64). Atherosclerotic plaque prone areas have a high concentration of Low density lipoprotein (LDL) distribution and the concentration distribution alone cannot explain the focal aggregation of LDL in the arterial wall. Due to the high clinical incidence of coronary atherosclerosis, it is of great clinical significance to study the distribution of hemodynamic parameters WSS and the selective accumulation of LDL in coronary arteries. Studies show that areas exposed to low WSS are more prone to atherosclerosis and more sensitive to high cholesterol levels and inflammatory mediators (5). Intense hypercholesterolemia and very low WSS are synergistic in favoring rapid atheroma progression (2). Chen et al. (65) reconstructed a three-dimensional (3D) computational model of the left coronary arterial tree from a patient-specific computed tomography angiography (CTA) image and investigated the transport of LDL from the lumen to the arterial wall using a numerical method of fluid-structure interaction (FSI). The results showed that early lipid-rich plaques were located at low WSS sites (66) and that the high level of LDL uptake coincided with low shear stress sites. Timmins et al. (67) demonstrate that, in patients with non-obstructive CAD, sectors subjected to low and oscillatory WSS demonstrated regression of total plaque, fibrous and fibrofatty tissue, and progression of necrotic core and dense calcium, which suggest a transformation to a more vulnerable phenotype. The inflammatory stimulus generated by the oxidation of accumulated lipids induces endothelial cells to capture monocytes that differentiate into macrophages, while the prolonged residence of LDL particles on the surface of low-WSS endothelial cells will upregulate pro-inflammatory transcription factors to initiate the endothelial response to pro-atherogenic stimuli, increasing the permeability of the endothelial cell surface to LDL and further promoting LDL aggregation there (41, 68). Early atherosclerotic plaque formation is rapid. Over time, high blood viscosity increases the time-averaged WSS (TAWSS) on the arterial surface, which directly affects the transport of LDL within the vascular lumen. Also, the thickened vessel wall inhibits the entry of LDL into the arterial wall, leading to slower plaque growth in later stages. Hartman et al. (64) found a dose-dependent relationship between lipid content and high WSS exposure. Most areas containing high lipids coexisted with high TAWSS, and plaque instability was also shown to be associated with high TAWSS.

### Effect of WSS on vascular inflammatory response

The inflammatory response, as the initiator of atherosclerosis, predisposes to a marked pathological expression of certain vascular proteins (69). Endothelial junctional adhesion molecule-A (JAM-A) is an important effector molecule that directs inflammatory cells to atherosclerosis-prone sites, and areas of disturbed blood flow show that local enrichment of JAM-A promotes the reassembly of monocytes into the arterial wall (70). Other findings suggest that disturbed blood flow increases the interaction between leukocytes and endothelial cells in flowing blood, promoting a tendency for inflammatory cells to accumulate in areas prone to atherosclerosis (71). Chatzizisis et al. (72) demonstrated that low WSS failed to induce ECs alignment in the direction of flow, and the coverage of vascular endothelial cells in this segment was reduced, which was prone to trigger a variety of inflammatory events, including increased permeability, ROS generation, NF-κB activity, and expression of receptors and cytokines that recruit leukocytes. Chronic recruitment and activation of leukocytes triggers a sustained inflammatory response that exacerbates plaque growth and encroaches into the vascular lumen (73). Accumulation of oxidized LDL and subsequent chemokine release will further stimulate leukocyte influx in plaques and induce Notch1 to express macrophages, which makes macrophages tend to be inflammatory M1 type, and further stimulate the production of proinflammatory signals and cytokines (73). In addition, low WSS also affects the expression of inflammatory factors by targeting NF-κB, MAPK pathway and other mechanisms. Low WSS activates the JNK1-ATF2 transcriptional program to promote NF-κB expression and promotes NF-κB activation by inducing positive regulators such as Toll-like receptors, inhibitor of kB kinase 2 (IKK2), and reactive oxygen species (ROS) (5). Low and oscillatory WSS stimulates endothelial cell inflammatory responses by expressing adhesion molecules and cytokines *via* nuclear factor NF-κB (74). Increased expression of IL-6 and CRP in low WSS regions indicates enhanced proatherogenic inflammatory activity (75, 76). Oscillatory shear stimulates mononuclear leukocyte adhesion and migration into the arterial wall (77). Monocytes display unique forward and backward motion in oscillatory shear, undergoing rolling, binding, and dissociation with other monocytes (78). Furthermore, the dramatic increase in WSS creates an environment for further development of atherosclerosis. Some *in vitro* and ex vivo studies have shown that acute high WSS is related to the activation of proinflammatory factors in endothelial cells, which has been identified as the most potent inducer of cytokine release. This may be mediated by signalling pathways *via* mitogen-activated protein kinase (MAPK) and nuclear factor-κB (NF-κB) (79). In vitro studies using human umbilical vein ECs (HUVECs) have demonstrated that acute exposure to high shear stress can lead to early activation of ERK1/2 and p38 (80). High WSS segments coexisted with increased macrophage levels and plaque rupture (41).

### Effect of WSS on vascular adhesion molecules

The expression of cell adhesion molecules induced by shear stress is highly dependent on the exact nature of the applied shear stress (6). High expression of biomarkers such as ICAM-1, VCAM-1, MCP-1 and E-selectin is a marker of endothelial inflammatory phenotype in atherosclerotic susceptible areas (27). Under activation of pathological shear stress, integrins increase their affinity towards ligands. As a result, the cell adhesion molecules (CAMs) undergo a conformational change and control the adhesion of monocytes to ECs (81). Shear-dependent expression of CAMs *via* NF-κB, such as ICAM-1 (82), VCAM-1 (83), MCP-1 (78)and E-selectin (77) under low and oscillatory WSS is increased, which affects leukocyte adhesion to activated endothelial cells and contributes to the early formation of atherosclerotic lesions. The adhesion of leukocytes from flowing blood to the intima of blood vessels involves binding between integrin receptors and their endothelial ligands. Subsequently, attached monocytes migrate to the inner membrane and, upon appropriate cytokine activation, transform into active macrophages and phagocytose LDL to develop into foam cells (6). VCAM-1 is involved in the rolling and adhesion of monocytes, and the expression of VCAM-1 and ICAM-1 in aortic regions with low WSS was demonstrated to be increased in ApoE^−/−^ mouse models (84). A colocalization of VCAM-1 and ICAM-1 with low WSS is in with the inner curvature of the ApoE^−/−^ mouse (21). Sustained high expression of VCAM-1 at low shear stress significantly increases the adhesion level of monocytes and causes monocyte aggregation (6). Chiu et al. (82)studied the effect of shear stress (20 dynes/cm^2^) on endothelial cell function induced by tumor necrosis factor (TNF). The results showed that shear stress played different roles in regulating the expression of cytokine induced adhesion molecules in endothelial cells. Shear stress upregulated ICAM-1 mRNA and protein expression induced by TNF, while VCAM-1 and E-selectin expression levels were decreased. This difference may be due to the inconsistent spatial acuity by which ECs respond to a gradient in shear stress, as shown by Suo et al. (21) *in vivo* study showed that VCAM-1 expression was prominent in the aortic arch but almost absent just millimeters away, and that VCAM-1 was low compared with the static region at high shear stress, even though NF-κB was uniformly activated (85). MCP-1 has been demonstrated in human atherosclerotic lesions and in plaques induced by animal models (86). OSS (oscillatory shear stress) has been shown to induce MCP-1 activity (87), and a low WSS environment can induce sustained activation of the MCP-1 gene in endothelial cells (88). The initial action of low WSS on static endothelial cells causes transient activation of Ras GTPase and MAPK, leading to transient expression of the MCP-1 gene. However, persistent low WSS decreases Ras activity and MCP-1 expression (27). The concentration of MCP-1 is affected by the WSS magnitude, while the transport of MCP-1 highly depends on the directionality of WSS vectors as well as the WSS LCS (29).

### Effect of WSS on activated platelets

Platelet activation and accumulation at the site of vascular injury play a key role in thrombosis, and the supraphysiological range of WSS formed by disturbed blood flow at the luminal stenosis can regulate platelet adhesion and accelerate platelet activation and thrombus growth (89). Shear stress changes and degree of stenosis are associated with intracoronary platelet P-selectin upregulation and platelet-monocyte aggregation (90). Local high WSS contributes to plaque thrombus formation (91). In coronary arteries with severe atherosclerotic lesions, high WSS enhances platelet activation and subsequent aggregation and may contribute to plaque instability or even plaque rupture by eroding the fibrous cap (42, 92). In a study that linked the plaque components imaged using MRI with CFD simulation of WSS, Groen et al. (93) proved that plaque ulceration occurred at a location exposed to high WSS. Therefore, high WSS is usually associated with platelet activation and plaque rupture (94). In vitro studies have also shown that arterial thrombosis occurs in the face of pathologic high WSS, and platelets in the high WSS region of the stenotic larynx produce rapid and firm binding without prior activation. At this time, platelet activation, adhesion and aggregation are the strongest (95). However, in the Al-Tamimi et al. (96) study, exposure to platelets with high WSS can induced GPVI cleavage, down-regulated GPVI expression, and decreased platelet reactivity to subcutaneous proteins, including collagen, under pathological shear conditions. Shear-induced GPVI shedding may be a novel protective mechanism for down-regulating platelet adhesiveness and reactivity, and increasing plasma sGPVI under atherothrombotic conditions. The different effects of high shear stress in different situations may be caused by the use of different research methods (pure vascular modeling to simulate coronary arteries or experimental studies) or the inconsistencies in the intervention parameters applied in the study subjects (virtual coronary arteries or human platelets). The flow rate in the area of low WSS is low, resulting in the continuous aggregation of blood cells and platelets in these locations. Under the action of condensing enzyme, blood cells and platelets in these locations continue to grow, which further aggravates the vascular stenosis in the separation area. Extensive exposure of the subcutaneous matrix due to significant erosion downstream of the plaque at the site of vascular stenosis triggers platelet adhesion in this area again and promotes the release of growth factors that induce pro-fibrotic responses, such as SMCs proliferation and matrix synthesis (97). Nesbitt et al. (98) revealed that discoid platelets preferentially adhere in low-shear zones at the downstream face of forming thrombus by hemodynamic analysis. Platelet aggregation appears to be affected by the magnitude of the shear gradient. When exposed to decelerating shear stress, the strength and stability of discoid platelet aggregates increase, thereby promoting sustained thrombus growth.

### Summary and prospect

**Figure 2:**
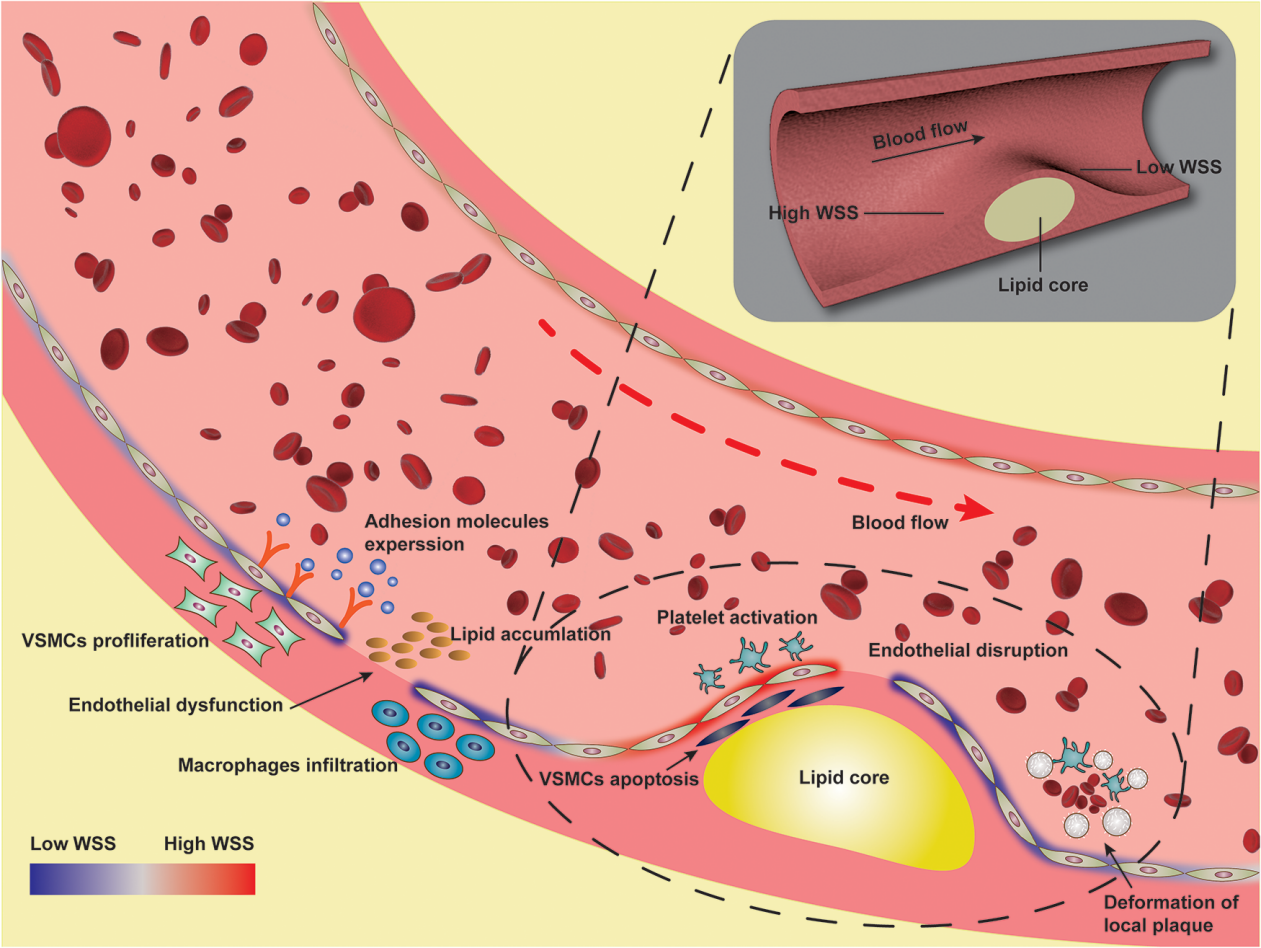
Illustration of pathological mechanism of wall shear stress involved in atherosclerosis.

In summary, WSS plays an important role in the occurrence and development of atherosclerosis. The complex blood flow in arteries forms rich WSS vectorial features (99), including the newly proposed WSS topological skeleton to identify and classify the WSS fixed points and manifolds in complex vascular geometries. CFD modeling represents one of the attempts to simulate these complex hemodynamic conditions to investigate the impact of WSS on atherosclerosis. Low WSS is the factor of early plaque formation, and high WSS promotes the transformation of plaque to high-risk phenotype, that is, low WSS induces the occurrence and development of plaque, while high WSS is related to plaque instability. WSS acts as a link between blood flow dynamics and the biology of various cardiovascular diseases (99). Low WSS and high WSS are involved in the loss of physiological flow alignment of endothelial cells, the increase of low-density lipoprotein accumulation, the proliferation and apoptosis of smooth muscle cells, the high expression of inflammatory factors and adhesion molecules, and the abnormal activation of platelets, which promote the occurrence and development of atherosclerosis. Overall, WSS can potentially provide insight into the onset of initial lesions and progression to more advanced lesions, playing a crucial role in regulating hemodynamically directed atherosclerotic vascular disease. Understanding the detailed mechanisms by which WSS regulates hemodynamically directed vascular disease will help us elucidate the pathogenesis of atherosclerosis and develop potential therapeutic strategies for atherosclerosis, as shown in Figure 2. The current clinical detection methods can assist in the secondary prevention after the occurrence of lesions, while more prognostic research is needed in the primary prevention process. The regulatory role of WSS in atherogenesis remains to be investigated. Combining clinical imaging techniques with CFD allows for detailed examination of local morphological and biomechanical characteristics of atherosclerotic lesions to identify more effective WSS-based hemodynamic indicators, so as to close the gap of knowledge currently limiting the use of WSS as a bio-marker for diagnostic and prognostic purposes. Ultimately, WSS will be used as a biomarker of vascular status and atherosclerotic lesion burden to identify early plaque and monitor plaque stability, and provide a rationale for a paradigm shift towards pre-emptive treatment strategies.

## Author contributions

WJ, LW and MZ: conceptualization; MZ and YY: writing-original draft; MZ, RC and XL: writing-review and editing; YH and LG: resources; ZM: validation. All authors contributed to the article and approved the submitted version.
